# Analysis of Deep Learning Techniques for Dental Informatics: A Systematic Literature Review

**DOI:** 10.3390/healthcare10101892

**Published:** 2022-09-28

**Authors:** Samah AbuSalim, Nordin Zakaria, Md Rafiqul Islam, Ganesh Kumar, Norehan Mokhtar, Said Jadid Abdulkadir

**Affiliations:** 1 Department of Computer and Information Sciences, Universiti Teknologi PETRONAS, Seri Iskandar 32610, Perak, Malaysia; 2 Data Science Institute (DSI), University of Technology Sydney (UTS), Ultimo, Sydney 2007, Australia; 3 Dental Simulation and Virtual Learning Research Excellence Consortium, Department of Dental Science, Advanced Medical and Dental Institute, Universiti Sains Malaysia, Bertam, Kepala Batas 13200, Penang, Malaysia

**Keywords:** dental informatics, dental practice, health informatics, dental diagnosis, deep learning

## Abstract

Within the ever-growing healthcare industry, dental informatics is a burgeoning field of study. One of the major obstacles to the health care system’s transformation is obtaining knowledge and insightful data from complex, high-dimensional, and diverse sources. Modern biomedical research, for instance, has seen an increase in the use of complex, heterogeneous, poorly documented, and generally unstructured electronic health records, imaging, sensor data, and text. There were still certain restrictions even after many current techniques were used to extract more robust and useful elements from the data for analysis. New effective paradigms for building end-to-end learning models from complex data are provided by the most recent deep learning technology breakthroughs. Therefore, the current study aims to examine the most recent research on the use of deep learning techniques for dental informatics problems and recommend creating comprehensive and meaningful interpretable structures that might benefit the healthcare industry. We also draw attention to some drawbacks and the need for better technique development and provide new perspectives about this exciting new development in the field.

## 1. Introduction

The use of information technology (IT) in healthcare practice and research is a global goal for many nations [1]. In the last fifty years, IT capabilities have advanced dramatically. Several advancements have enabled new and beneficial applications of IT in the medical field. The interdisciplinary discipline of medical informatics (MI) combines software, computer science, medicine, information science, statistics, cognitive sciences, and mathematics [2]. This field’s task and mission is to reduce costs while improving health care services, and also care errors by using concepts, tools, methods, software techniques, and modeling [3,4]. MI can be considered of as a subdiscipline of dental informatics (DI); hence MI has some influence on DI’s progress. Despite the similarities between DI and MI in medical research, it is important to perform separate studies that are specifically focused on DI. Information science and computer applications improve dental research, practice, management, and education, which has enormous potential in the relatively new field of DI. The use of computing in dentistry is only one aspect of DI. The initial practitioners of DI defined their strategy as the use of information science to address medical issues. More recent publications have described MI as a cascade from analysis to effect. A four-part structure is suggested by one previous study. The four parts are: formulation of the system development, evaluation, medical model, and system installation and modification. The inherent challenges at each phase in this procedure are the biggest challenge for much of DI [5]. Sadly, most dentists are unaware of what DI is, what its objectives are, what it has accomplished so far, and how they might participate in it [6]. DI may provide a variety of tools and applications for the purpose of clinical practice of the oral diagnosis of illnesses, the contraindications, indications and prescription of particular medications to patients with particular problems, and other areas. Technological advances have made a significant contribution to the introduction of innovative conservative techniques in several medical branches. These procedures stand out for their significant decrease in operating time and invasiveness as well as their significant enhancement of patients’ psychological and physical comfort. Likewise, as in other industries, dentistry has incorporated the digital workflow in a variety of sectors, including treatment planning, designing, prototyping, implant surgery, and the creation of specialized prosthetics.

Digital dentistry technology, especially in recent years, have been crucial in altering patient interactions and developing creative and all-encompassing restorative approaches [7,8]. Cone beam computed tomography (CBCT) has made it possible to improve diagnostic datasets through digital radiography and data collection [9]. Additionally, the implementation of CAD–CAM technology and 3D manufacturing processes (such as stereolithography, 3D printing, etc.) and modern treatment modalities for procedures involving dental implants, such as computer-guided implant surgery, can be introduced owing to implant dentistry [10]. In comparison to conventional surgical approaches, this strategy resulted in considerable improvements and simplification, increasing implant location accuracy while also enhancing patient comfort and compliance [11,12]. By utilizing mixed reality (MR), virtual reality (VR) and augmented reality (AR) to improve students’ learning and clinical training, contemporary digital technologies have the ability to fundamentally alter dentistry on both an educational and clinical level. These technologies could be helpful tools for dental doctors in their work. Significant improvements in computational techniques for data analysis and processing are being driven by subjects including computer science, information science, statistics, biomedical informatics, and others. For example, text mining, data analysis, medical diagnosis, and hypothesis generation [13,14] all employ machine learning (ML), which was originally a relatively unexplored area of artificial intelligence (AI). More advanced ML algorithm-based techniques have recently been used to improve oral health [15]. One of these methods is generally recognized as being DL, which has proven to be effective in both disease prediction and prognosis. Numerous publications on oral disorders employing DL have been published during the past few years [16,17,18,19]. DL algorithms are effective at handling the difficulties and complexities of oral disease automated diagnosis. There have been numerous review studies on the detection and classification of oral disorders to date, but very few have been capable of offering a clear path forward for scholars. Even though this research provided an excellent literature review of dental disorders and applications, they should have covered more DL-related topics. The majority of the review studies [20,21,22,23,24,25,26,27,28,29,30,31,32,33,34] dentistry primarily focused on classic ML methods or generic artificial neural networks (ANNs), when feature extraction for diagnosis is required [35], and where feature extraction is involved for diagnosis. They could not address emerging DL architectures on dental disease diagnosis, such as generative adversarial networks (GANs) [36], extreme learning machines (ELMs) [37], or graph convolutional networks (GCNs) [38,39], etc. Although several review publications for dental medical imaging techniques and digital technologies are accessible [7], they are unable to cover all imaging modalities utilized in the identification and classification of dental illnesses. Additionally, they failed to provide an exhaustive summary of the merits and demerits of earlier research, making their analysis of learning-based deep approaches unclear. Because of this, this study provides a solid foundation for a thorough and critical examination of modern DL-based digital dentistry technology and dental disease diagnostics. Based on their popularity, we chose studies from 2017 to 2022 to conduct this study. In this study, the researcher advocated a systematic review approach that will assist upcoming scholars in figuring out the general framework of a dental diagnostic based on DL. This research provides a detailed picture of the deep neural network designs used in several DI areas to identify dental diagnostics. Imaging approaches for identifying and categorizing dental diagnostics are also covered in this study. Lastly, this systematic literature review (SLR) points future scholars toward a number of open research challenges and opportunities. This study, in our opinion, provides a valuable framework for scientists who work on classifying medical images, who may be involved in the switch to DL-based dentistry diagnostics, and who use various medical images.

The following is the study structure of the current paper. Section 2 includes a three-step demonstration of research methodology that includes planning, executing, and reporting the review. The conclusions of the selected publications, research topics, customary practices, data formats, and performance approaches are covered in Section 3. The scientific contribution of this review, management implications, and academic implications are addressed in Section 4, along with a discussion of current remedies. The restrictions and potential research directions demonstrated in Section 5, and Section 6 includes the conclusion in its final form.

## 2. Research Methodology

The philosophy from references [40] was followed in this SLR. There are three phases to the research process. The phases of defining research topics, designing, and verifying review methods are addressed in the initial planning phase. In the second phase, data extraction, information synthesis, and the finding and selection of pertinent research are discussed. Writing and validating the SLR is covered in third part. Figure S1 (kindly refer to the Supplementary File) shows how all three steps progress.

### 2.1. Plan Review

The crucial research questions and the creation of review protocols are laid out in this initial stage of the research process by using the right searching techniques.

#### 2.1.1. Research Questions

The current paper aims the following research questions posed in this SLR, and potentially all of them are later addressed with appropriate solutions.

**RQ #1: What are the existing DL techniques used in dental practice?** The study objective is to determine the relevance of digital imaging methods employed in the dentistry profession by researchers and clinics for their models, frameworks, or applications. DI deals with a wide range of data that is hard to monitor, interpret, and extract useful information from.

**RQ #2: Which categories of DI are adopting to use of the DL techniques?** This research issue is related to the categories of DI. The goal of this research is to have a thorough understanding of the procedures used in DI. The purpose of this study is to look at the applications, frameworks, and models that leverage DL approaches solve DI issues. Furthermore, phrases like “data informatics”, “deep learning”, “dentistry”, and “dental data” were used to acquire pertinent data in a novel way.

**RQ #3: Which type of images and datasets are used in dental informatics along with DL techniques?** The goal of this research question is to discover picture data, datasets, and approaches for dealing with DI problems. For ongoing study and future representation, the image, datasets, and relevant data can be used. It can also be employed for information retrieval and predictive analysis. As a result, detailed approaches for information retrieval, data formats, and performance metrics are discussed later.

**RQ #4: What are the performance measurement techniques that are used to measure the performance of DL techniques in dental informatics?** The purpose of this research question is to discover the DL model, framework, and techniques’ performance used in DI. The images and other relevant formats of data that are used in DL models will be reviewed and reported.

#### 2.1.2. Review Protocols

The review protocol’s development and authentication support the selection of relevant keywords when looking for relevant articles and literature sources.

#### 2.1.3. Searching Keywords

The researchers tried to narrow the search to the most relevant particular keyword to ensure that the evaluation closely covers DL approaches for DI and the following steps were taken out.

- Taking the key terms from our research questions and extracting them.
- Referring to the terms by various names.
- Adding keywords from pertinent publications to our search terms.

To find the most immediately pertinent works in the literature, the researchers utilized the primary alternatives and added the “OR operator” and “AND operator”, as shown in Table 1.

#### 2.1.4. Literature Resources

The selection of relevant publications for primary review studies was obtained from the databases Web of Science, Scopus, ACM Digital Library, Springer, Science Direct, and IEEE Explorer. Databases such as ISI and Scopus indexed papers and certain publications from prominent conferences containing the most comprehensive coverage of quality articles on our subject. By using the sophisticated search options given by each of these databases, the search phrase was created. Our search included the years 2017 through 2022.

### 2.2. Conduct Review

This section includes the pattern of conducting the review by using research questions, keywords, and protocols as a guide. According to Table S1a,b, this phase is mainly concerned with article inclusion and exclusion.

#### 2.2.1. Study Selection

**Figure 1:**
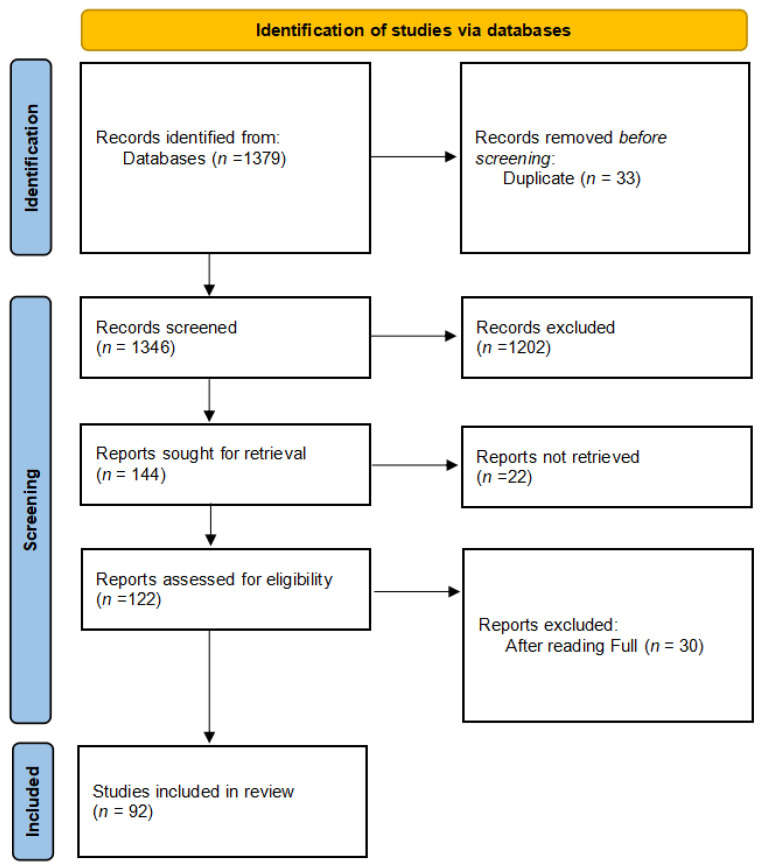
Flowchart review process and identifying the number of relevant studies.

Figure 1 depicts the entire process of study selection. The web search yielded a total of 1355 articles. A total of 155 articles were short-listed after filtration by using title, keyword, inclusion, and exclusion criteria. Table S1a,b reveal the inclusion and exclusion criteria. There were 33 articles from other fields, such as biology, illness, and other languages, that were replicated in other databases, and 22 articles from different concepts, such as visualization, prediction, and other languages. After undergoing the whole article reading, 30 items are deleted from the list.

The criteria for choosing related research articles based on keywords are described in Table S1a,b. Repeated research articles and those that may not address all of the research questions were omitted.

The quality checklist criteria for study evaluation are included in Table S2. The questions are primarily meant to assist in the selection of studies that are more relevant, thorough, and comprehensive in nature.

#### 2.2.2. Data Extraction

The researcher has used the data-extraction techniques listed in Table S3 to gather the data required to address our research questions and contributions.

#### 2.2.3. Information Synthesis

The extracted data were consolidated at this point in order to respond to the study questions. The narrative synthesis approach was employed to answer our study’s questions. As a result, we presented our findings by using tables and charts.

### 2.3. Report Review

Our four research questions were addressed by using data that was taken from the original studies.

## 3. Results

Approximately 79 studies were included in the evaluation. A total of 40 studies in total were related to the DL techniques that are being used in the dental practice, and 39 were used to answer the categories of DI that are using the DL techniques. However, the same studies (i.e., studies used for DI using DL techniques) contributed to answer the question related to the type of images used to evaluate the DL techniques in dentistry. A total of 56 studies were evaluated to find the performance measurement techniques used to evaluate the DL techniques in dentistry, as shown in Table 2.

Figure S2 illustrated the amount of research on DL carried out in the domain of dentistry per year. As clearly shown in the figure, there is a significant increment in the amount of research carried out in this domain. There were only three research pieces found in this domain in 2017; however, the number significantly increased in the coming years to 9, 11, 21, 29 and 6 in the years of 2018, 2019, 2020, 2021 and early 2022, respectively.

### 3.1. What Are the Existing DL Techniques Used in Dental Practice?

A total of 40 studies were reviewed in this SLR discussing the DL techniques used in dental practice. The following subsections explain DL techniques used for DI.

#### 3.1.1. Artificial Neural Networks (ANNs)

Dental disease diagnosis with ANN is a very active study field in medicine right now. The first research studies in the literature used ANN based upon radiographic images as an alternative for radiation-related caries (RRC) detection to predict RRC lesions with a 99.2% accuracy [41] rate. This technique demonstrates that further research on RRC prediction and detection might enhance dental treatment for head-and-neck cancer (HNC) patients. Li et al. [42] developed a segmentation architecture for detecting areas with five frequent gum disorders in their study. The proposed semantic segmentation architecture is based on the DeepLabv3+ network with Xception and MobileNetV2 as the backbone. Most of the gum inflammation region may be correctly divided into four or five groups by using the suggested segmentation methodology. Laplacian filtering, statistical extraction of features, morphological operations, window-based adaptive cutoff, and backward-propagation neural network (BPNN) are all parts of the diagnostic system proposed in [43]. The BPNN algorithm is utilized in this study to classify whether a tooth surface has dental caries (DC) or is normal. This model based on BPNN can predict DC more accurately. Zanella–Calzada et al. [44] aimed to investigate the determinants of oral health based on dietary and demographic characteristics by using dense ANN. ANN has a learning method which is called the extreme learning machine (ELM), a single hidden-layer feedforward neural network (SLFN) [45]. Rochman et al. [46] applied the ELM approach to estimate the number of patient visits in Dental Poli. ELM generates prediction output with a 0.0426 low error rate. Combining the Hu moment invariant (HMI) method with ELM, [47] devised and implemented a unique classification technique for CBCT images of teeth. The results showed that the devised methodology is better compared to a statistically significant ANN. Another study uses an algorithm that combines principal component analysis (PCA) evaluation and extreme learning to apply ML to the categorization of teeth. Overall, the research was able to categorize molars, premolars, canines, and incisors with an accuracy of 79.75% [48]. Li et al. [49] developed another automated technique for categorizing tooth kinds on dental pictures by utilizing ELM and a gray-level cooccurrence matrix (GLCM) [50]. Experiments indicate that the suggested technique is more sensitive and precise than the naïve Bayes and the wavelet energy. The suggested method has the benefit of achieving higher accuracy in classification without requiring exact segmentation of the teeth. Table 3 summarized the studies that apply ANNs.

#### 3.1.2. Recurrent Neural Networks (RNNs)

RNNs are an ANN algorithm that discovers time-dependent correlations between input data by merging the historical data stored in hidden layers also with current input value. Alarifi and Al Zubi [51] examine the dental implant therapy consecutive measure by using a memetic search optimization and genetic scale recurrent neural network (MSGSRNN). Due to its low error rate, the described approach requires a greater degree of precision (99.25). Kumari et al. [52] implement a novel hybrid DC segment by using ResneXt-RNN and FOC-KKC. In a pre-processing step, noise filtering and contrast-limited adaptive histogram equalization (CLAHE) are conducted. Caries are further segmented by using fused optimal centroid K-means with K-mediods clustering algorithm (FOC-KKC). Compared to traditional approaches, the proposed MResneXtRNN and novel segmentation algorithm for caries prediction have shown improved performance. Long short term memory (LSTM), out of the suggested variations of RNN, has been a cutting-edge model for several situations in the last few years. In 2021, Singh and Sehgal [53] proposed a novel LSTM model by using CNN for diagnosing detecting DCs in periapical dental pictures. A CNN was used to extract features from periapical dental pictures, and then short-term and long-term dependencies were calculated. This study has studied the G.V. Black categorization with the categorization of DC categories as its primary objective. The experimental examination of optimum CNN-LSTM displayed competitive performance in the categorization of dental images. Table 4 shows the studies that used RNN technique.

#### 3.1.3. Convolutional Neural Networks (CNNs)

In the dentistry industry, CNN diagnosis accuracy approaches human skill levels [44,54]. Radiographs are commonly utilized as image inputs for CNNs to diagnose diseases in dentistry. Dental disease detection has been taught specifically on bitewings [43,55], periapical radiographs [56], and panoramic X-rays [57]. Periapical images are highly beneficial for diagnosing possible caries, periodontal bone loss, and periapical issues [58]. Lee et al. [56] adopted a pre-trained GoogleNet Inception v3 network for the diagnosis and prediction of dental caries by using 3000 periapical radiographs. The premolar and molar areas, as well as the premolar–molar region, demonstrated exceptionally high accuracy of 82%, 89%, and 88%. Similarly, Ref. [57] suggested using CNN to identify caries in the third molar by using a clipped picture of the third molar from a panoramic radiography image. Only simple arrangements with little overlaps are possible to see in bitewing pictures of the crowns of posterior teeth [59]. In dentistry, panoramic radiographies are frequently employed because they allow for the screening of a significant anatomical area with only a little amount of radiation exposure [60]. Near-infrared light transillumination (NILT) is more beneficial when taken in youngsters or at shorter intervals, as in high-risk persons. Several in vivo and in vitro investigations examined the accuracy of NILT, concluding that it is appropriate for identifying both primary and secondary caries lesions [61,62]. By using transfer learning, a CNN was utilized to categorize dental disorders such as dental caries, periapical infection and periodontitis. The researchers classified dental disorders by using the pre-trained model VGG16, attaining an overall accuracy of around 88% throughout radio visiographic (RVG) X-ray scans [16]. A caries probability map is also created by using a CNN, and crown areas are retrieved by using optimization techniques and an edge-based level set approach to segment crown regions [63]. The research depicts that the proposed system achieves a higher performance. In the CNN model, periapical radiographs performed exceptionally well in diagnosing dental caries [64,65,66]. Dental image diagnosis information was integrated into an automated, simplified dental image analysis technique by employing a CNN. A method for evaluating the quality of an automated root canal treatment was presented by Yang et al. [67]. The investigators used a dental scan-based automatic apical foreman-area recognition technique for the root canal filling therapy. This study’s authors used a labelled dataset of periapical radiography images taken both before and after treatment. This allowed the authors to identify the apical foreman and the area around it by utilising the filling area attained a 0.749% F1 score. The deep CNN algorithm proved effective in determining PCT diagnosis and prediction. To assess the potential utility and accuracy of a computer-assisted detection system based on a deep CNN algorithm for the diagnosis and prediction of periodontally damaged teeth (PCT) was developed. The deep CNN algorithm exhibited a greater diagnostic accuracy for recognizing PCT in premolars than in molars, according to the current study [68]. They discovered that the network had comparable results to a skilled periodontist. In 2019, Al Kheraif et al. [69] handled the critical area segmentation of dental pictures by using CNN and deep learning and showed an improvement in accuracy of 97.07%. Typical segmentation techniques, such as common-edge detection, may fail to remove noise and damaged pixels from input pictures. The CNN technique was utilized on panoramic radiography to identify maxillary sinusitis and explain its diagnostic performance. Murata et al. [70] assessed the diagnostic performance of a DL system by employing panoramic radiographs to diagnose maxillary sinusitis. It was compared to the performance of two residents and two radiologists. The system’s diagnostic efficiency was comparable to that of radiologists. However, AI performed better than dental residents. On panoramic dental radiographs, deep CNNs were utilized to identify apical lesions [71] and periodontal bone loss (pBL) [72]. For analyzing pBL on panoramic radiographs, a custom CNN trained on a small number of picture segments exhibited at least an equivalent discriminating skill as dentists. The proposed method using deep DCNNs gives the classification accuracy of 0.81 (0.02). By utilizing ML-based technology, dentists’ diagnostic efforts when using radiographs might decrease [72]. From panoramic dental radiographs, a hybrid CNN and SVM technique was utilized to detect apparent dental caries/periapical infection, changed periodontal bone height, and third molar impactions [73], whereas panoramic dental radiographs were used for teeth recognition [74,75], classification of dental problems [74] and tooth decay [76] in X-ray images. A two-staged attention segmentation network (TSASNet) [77] was developed to locate and categorize teeth in radiographs. First, the attention model is used to establish the approximate placement of the tooth. Following this, the exact tooth boundaries are identified with a precision of 96.94% by using a deep convolution network. The dental and background segmentation methods were employed in a dental X-ray for automated tooth and backdrop segmentation by utilizing the U-Net convolution network DL approach [78]. The experimental findings demonstrate that the suggested U-Net convolutional network achieves a classification precision of 97.61% on average.

In the early phases, Sobel edge detection with deep CNN was employed to forecast cavities. The gradient direction of Gx and Gy is determined by using the Sobel edge-detection method [79]. The algorithm executed the Sobel edge identification by using DCNN to detect the cavities with the efficient accuracy of 96.08%. Automatic feature segmentation of common radiographic abnormalities, including alveolar bone loss, interradicular radiolucency and dental caries was achieved by using DL-based networks such U-Net, Segnet, XNet, U-Net +, and Densenet [80]. CNNs were also used to identify locations in periapical exams based on the presence of periodontal bone loss [81] and other dental disease detection [82] indicators. Tajinda et al. integrated segmentation and classification tasks for grading periodontitis from periapical radiography images to create the hybrid network for periodontitis stages from radiograph (HYNETS) end-to-end DL network. By combining segmentation networks and a classification network, HYNETS uses a multi-task learning technique to provide a complete, interpretable solution with extremely accurate and reliable results [83]. Together with data pre-processing and augmentation techniques, Szu-Yin Lin and Hao-Yun Chang have created an innovative and effective two-phase DPR detection and methodology to help dentists in diagnosis by using advanced DL algorithms. Orthodontics, dental restoration, endodontic therapy, implant, impaction, and dental prosthesis are among the dental problems that are instantly detected [84]. To categorize the teeth positions by using a proposed correlation module that makes use of the information between teeth positions, Zhang et al. [85] employed DL methods and used a particular label creation methodology to partition the teeth classification job before using a multi-task CNN. The results of these approaches are satisfactory for the purposes intended. For the automated diagnosis [86] of dental caries based on periapical pictures, an MI-DCNNE model, developed by Imak et al., is used as a multi-input deep CNN ensemble. The proposed MI-DCNNE technique was more successfully implemented by using a score-based ensemble approach with 99.13% accuracy score [87]. Several researchers demonstrate a DL strategy for identifying and localizing dental lesions [88] in TI images automatically and dental carries in NILT images [89,90] and on the children’s first permanent molar [91]. Their research shows that using a DL technique to analyze dental photos can improve caries detection speed and accuracy, as well as complement dental practitioners’ diagnosis and improve patient outcomes [88]. Rana et al. present an automated approach for segmenting dental images pixel by pixel and successfully distinguish gingival inflammation from healthy gums. Oral pictures are used to segment gingival diseases. The automated technology uses intraoral pictures at point-of-care settings to assist in avoiding severe periodontal disease and tooth loss by detecting gingival inflammation early in patients [92]. A Mask R-CNN model can identify and categorize dental caries throughout the whole 7-class ICDAS scale [93]. They used deep learning to diagnose dental cavities in intraoral pictures acquired by intraoral cameras, attaining precisions of 0.667, 0.889, and 0.778 in the most frequent, worst classes and centroid pixel class. Tanriver et al. [94] evaluated the usefulness of image-processing technologies in the detection of oncology. With a second stage classifier network, a two-stage DL model was suggested to identify oral cancer and categorize the discovered area into three categories of benign oral, and possibly malignant carcinoma. By using the expert standard as a reference, Schlickenrieder et al. assess a CNN trained to identify and categorize fissure sealants from intraoral images. According to this investigation, a trained CNN recognized sealant intraoral pictures with 98.7% agreement with reference judgments [95]. The DL method called YOLOv3 was suggested by Takahashi et al. [96] for identifying dental implants and tooth restorations. However, the effectiveness of that method was limited in identifying tooth-colored prostheses. To identify white spot lesions in dental pictures obtained with a digital camera, Askar et al. used DL approaches. They demonstrated satisfactory accuracy in identifying white spot lesions, particularly fluorosis [97]. Table 5 shows the studies that used the CNN technique.

#### 3.1.4. Generative Adversarial Networks (GANs)

With exceptional performance, GAN can train the generative model of any data distribution by using adversarial approaches. Since its inception, GAN has attracted a lot of attention due to its outstanding performance. The innovative adversarial learning concept of GAN permeates all facets of deep learning significantly, leading to a number of new research avenues and applications, which is especially important. Goodfellow et al. [98] introduced GAN to the DL space. As its name implies, GAN, a class of generative models, is trained in an adversarial environmental deep neural network. Kim et al. [99] used masks to remove the interdental space, and then GAN was used to recreate the edge outlines. The proposed method increased the precision to 0.004 mm when compared to separated scanning without interdental areas. Due to the masking of nearby normal structures, the size of the mask was, nevertheless, negatively correlated with the accuracy of the reconstruction. Kokomoto et al. [100] demonstrated the creation of full-color intraoral images by using progressive growth of generative adversarial networks (PGGAN), and they assess the quantity and visual quality of the produced intraoral photos according to paediatric dentists. Without raising any privacy issues, the obtained intraoral images can be used as instructional materials or as data augmentation for DL. Table 6 shows the studies that used GAN technique.

#### 3.1.5. Graph Neural Networks (GNNs)

Graphs are a type of data representation that is related to non-Euclidean, irregular domains. Several physical human operations create data that is contained in a graph form by default. Graphs by their very nature capture relationships between things, making them potentially highly helpful for encoding relational information between variables in these applications [101]. As a result, the extension of GNN into non-structural (unordered) and structural (ordered) contexts has received a lot of attention. In order to learn additional discriminative geometric characteristics for 3D dental model segmentation, Zhang et al. [102] offer a novel two-stream GCN capable of processing coordinates and normal vectors separately. Another method proposed by Zheng et al. [103] called TeethGNN is a graph-based neural network for semantic dental teeth segmentation. They introduced a novel two-branch architecture: a semantic branch to produce facet-wise semantic labels and an offset branch to predict an offset-to-centroid vector for each graph node. Although graph-based representations are becoming increasingly frequent in the medical arena [104,105,106,107,108,109], they are still uncommon compared to traditional DL methods, and their promise to solve a wide range of difficult medical issues has yet to be completely realized. Table 7 shows the studies that used GNN technique.

### 3.2. Which Categories of DI Used DL Techniques?

Approximately 39 research papers were studied to find various categories of DI using different DL applications in dentistry. The following subsections present various DI categories that used DL techniques.

#### 3.2.1. Computer Aided Design (CAD)/Computer Aided Manufacturing (CAM)

CAD–CAM is a newly developed scope of dental restoration and prosthodontics rehabilitation that employs CAD–CAM systems to design and fit a variety of dental restorations, including zirconium crowns, fixed bridges, dental implant restorations, orthodontic appliances, dental (inlays, veneers, onlays), and removable dentures (partial and/or complete) [110]. The epithelial dysplasia illness has been classified by using a CAD approach. The algorithm collects a wide range of features and qualities, then sorts them into two categories according to their relative importance [111]. Features were retrieved by using the oriented FAST and rotated BRIEF (ORB) method and classified with the support vector machine (SVM). Oral epithelial dysplasia classification accuracy was 92.8% using the suggested method. Chatterjee et al. [112] suggest a computer-assisted technique to diagnose oral pre-cancer/cancer using an oral exfoliative cytology. They used a combination of statistical features such as morphology, intensity, color, texture, and histogram for diagnosis of oral malignancy. They reported maximum recall of 94.58 % by using a random forest classifier. With 3D STL models of a die scanned from patients, AI displayed significantly good performance in forecasting the debonding probability of CAD–CAM CR crowns. This technology could be used to help dentists during or after restorative procedures, as well as in other troublesome cases, such root or die fractures [113]. Various approaches were proposed for 3D dental model segmentation and classification. To categorize mesh cells, Xu et al. [114] advocated reshaping hand-crafted geometric characteristics as 2D picture patches to train 2D convolutional neural networks. The mesh labeling approach achieves a level of precision of 99.06% (as measured in area) that is directly usable in orthodontic CAD systems, surpassing the accuracy of state-of-the-art geometry-based methods. A technique for segmenting and classifying teeth on 3D digital dental molds is proposed by Tian et al. [115], which employs sparse voxel octree and 3D CNN. This approach successfully segments teeth with an accuracy of 89.81%. Table 8 shows the studies that used CAD–CAM technique.

#### 3.2.2. Three-Dimensional (3D) Printing

Three-dimensional modeling applications in dentistry extend from oral and maxillofacial surgery, oral implantology, and prosthodontics to periodontology, endodontics, and orthodontics [116,117]. Tian et al. [118] present a pragmatic and scientific review of 3D printing technology in dentistry. Liu et al. [119] present a feature extraction approach that is an end-to-end DL for 3D printings of tooth models. The experiment has a 92.6% accuracy on the validation set. Due to the wide diversity of teeth in the dental model, there is also the difficulty of how to better utilize geometric aspects of the teeth, in addition to the necessity of manually marking a significant number of data samples.

#### 3.2.3. Electronic Dental Records (EDR)

EDR systems are extensively utilized in the dental practice and serve as an important resource for data-driven clinical decision-making research. Cui et al. [120] used electronic dental data to build a clinical decision support (CDS) model that predicts tooth-extraction treatment in clinical scenarios (EDRs). The model demonstrated a 96.2% of accuracy and proved to be a potent regressor and classifier, achieving ideal performance with structured data. Kang et al. [121] suggested utilizing ML to forecast a DC model in personalized medicine. The suggested approach, called DCP, employs DL models as well as several ML models. Random forest has achieved the highest performance compared to other machine learning methods, with an accuracy of 92%, an F1 score of 90%, precision of 94%, and a recall of 87%. Chen et al. [122] proposed a method for extracting data from Chinese EDRs for clinical decision support systems byy using natural language processing (NLP). They apply hybrid methods combining a keyword-based method and DL methods (word2vec and sentence2vec), and the resulting models have an F1 score of 88% and 83%, respectively. Table 9 shows the studies that used EDR technique.

#### 3.2.4. Cone Beam Computed Tomography (CBCT)

Endodontics, orthodontics, implant usage, oral surgery, and oral medicine have all benefited from the use of CBCT [123]. DL and AI have considerable potential for providing completely automated CBCT analysis, which can help decrease subjectivity and inaccuracies. This skill can also aid in the streamlining and expediting of healthcare processes. On dental cone-beam computed tomography (CT) images, a DCNN was used to classify tooth kinds. The use of a deep CNN with an AlexNet network architecture for tooth classification in dental CBCT pictures was researched by Miki et al. [124]. In addition to its potential utility in forensics, the seven-tooth-type categorization result has practical applications in the automated generation of dental charts. Sorkhabi and Khajeh [125] presented a 3D CNN approach to assess the alveolar bone density by using CBCT volumetric data, which may be used for classification of alveolar bone density. In addition, Jaskari et al. [126] investigated a DL technique for automated localization of the mandibular canals by using a CNN segmentation on a clinically varied dataset of cone beam CT volumes. Kwak et al. [127] have proposed using 3D U-Nets to identify and classify the mandibular canal in CBCT scans. A dental segmentation automated technique was used in the trials along with algorithms based on 3D, 2D, and 2D SegNet. A new set of 3D annotated mandibular photos was proposed by [128]. In 2020, Kim et al. built two multi-channel DL models to classify skeletal malocclusions on CBCT images [129]. The suggested models attained an overall precision greater than 93%. Orhan et al. created a smart algorithm built on U-Net structure to spontaneously recognize periapical diseases and quantify their volumes on CBCT images [130]. This technique attained a detect ability of 89% without any statistically significant differences comparing manual and automated volumetric estimates. Cui et al. [131] described a technique for the identification and segmentation of teeth from CBCT images using a 3D area proposal network with a learned-similarity matrix. Mask R-CNN was applied to recognize teeth in CBCT scans, and a Dice score of 0.9237 was achieved. By using a 3D convolution network, Chen et al. [132] extracted a single tooth from a small dataset (25 scans) of CBCT scans and achieved a structural similarity of 0.936 coefficient. Lee et al. [133] selected a U-Net architecture approach to segment teeth for implants by labeling all sections of two CBCT specimens and five slices of other samples. They developed a multi-stage training procedure, with each stage 491 expanding the distance between the teeth. Wang et al. assessed DL for multiclass CBCT image classification, which combines tooth and jaw bone (maxilla and mandible) segmentation at the same time [134]. To identify the distal root structure of the mandibular first molar on panorama diagnostic imaging, Hiraiwa et al. [135] employed a DL system (GoogleNet and AlexNet). Both DL algorithms performed diagnostics marginally better than highly trained radiologists. Dental panoramic radiography and CBCT scans based on a deep CNN (DCNN) were examined by Lee et al. [136] for the diagnosis and detection of odontogenic cystic lesions (OCLs), particularly periapical cysts, dentigerous cysts, and dontogenic keratocysts. An innovative AI system based on DL techniques was examined to ascertain the real-time performance of CBCT imaging diagnostic of anatomical landmarks, pathologies, clinical efficacy, and safety when employed by dentists in a clinical scenario [137]. Dental CBCT mandible segmentation using a unique end-to-end method based on shape-aware segmentation for mandible segmentation (SASeg) was proposed by Qiu et al. [138]. A mean mandible shape is used by SASeg’s prior shape feature extractor (PSFE) module, and recurrent connections preserve the mandible’s continuity structure. To categorise C-shaped canal morphology in mandibular second molars from CBCT volumes and to assess the effectiveness of three different architectures, a DL model was developed [139]. Various DL methods were used like U-Net, residual U-Net, and Xception U-Net architectures for image segmentation and classification of C-shape anatomies. Table 10 shows the studies that used CBCT technique.

#### 3.2.5. Finite Element Analysis (FEA)

Roy et al. [140] developed a unique technique for designing the form and geometry of dental implants by using ANN, FEA, genetic algorithms, and the desirability function in aggregation to achieve targeted microstrain. Lin and Su [141] use the finite element approach to conduct a biomechanical investigation of the influence of four typical occlusion circumstances on the various placements of dental implants. Another study suggested by Prati et al. [142] used an FEA to calculate the stress distribution created in the root dentine canal during the mechanical rotation of five distinct NiTi endodontic tools (FEA). Furthermore, Phanijjiva et al. [143] created a unique actual geometry of a complete tooth 3D model by utilizing a CT scan system and performed static structural assessments by using FEA.

#### 3.2.6. Virtual Reality (VR)/Augmented Reality (AR)/Mixed Reality (MR)

Li et al. [144] present an overview of the existing dental simulators on related technologies, benefits and drawbacks, methods of measuring efficacy, and future research possibilities. Gandedkar et al. [145] provide insight into the limitations of traditional education and investigate the existing and future uses of VR, AR, and AI in orthodontic teaching and research. Dyulicheva et al. [146] report the creation of a virtual reality simulator for dental offices that includes immersion in a VR scenario and simulation of tooth drilling. Dixon et al. [147] want to test the contemporaneous validity of the evaluation as well as the provision of qualitative feedback for cavity preparations by using VR dental simulators. The primary use of AR in dentistry is connected to overlaying digital information in the actual environment, essentially “enhancing reality”, and live communication systems between collaborators and patients via the exchange of photographs, videos, and 3D models. AR was originally utilized in dentistry for educational reasons as a technique by which to objectively evaluate pupils and provide immediate feedback [148]. Rao et al. [149] used ML and AR validation methods to improve 3D renderings of skeletal landmarks for instructing the students in orthodontic cephalometry’s science. Touati et al. [150] compare two unique AR communication tactics in dentistry. These tactics allow the user to rapidly test a virtual grin proposition by capturing a series of photos from various angles or by utilizing the iPad as an improved mirror. Monterubbianesi et al. [151] review the uses of VR, AR and MR in dentistry, as well as future digitalization problems, such as robotics and AI.

#### 3.2.7. Teledentistry

The use of teledentistry for distant consultation, treatment planning, dental screening, and diagnosis, and has been proven to be beneficial over the years. It has been shown to be equivalent to real-time consultations in places with restricted facilities access, among schoolchildren, and in long-term healthcare institutions [152,153]. Al-Khalifa and Al Sheikh [154] aimed to survey the Saudi dentists’ view of the benefits of teledentistry in enhancing dental practice and patient care. This study’s responses indicated that dental practitioners were prepared to use the teledentistry technique. Babar et al. [155] offer a data management approach for smart dental planning based on big data analytics. Teleconsultation, telediagnosis, teletriage, and telemonitoring are teledentistry modules with key tasks in dental practice [156].

### 3.3. Which Types of Images Are Used to Evaluate DL Techniques?

The studies used in RQ1 and RQ2 have been used to evaluate the type of images that are used to evaluate DL techniques. Different types of images were used by different researchers based on the techniques they used in DI. Radiographic images [16,41,43,56,63,64,65,67,68,69,70,71,72,73,74,75,76,77,78,79,80,81,82,83,84,85,87,157], near-infrared light transillumination (NILT) [88,89,90], intraoral images [66,86,91,92,93,95,96,97,158,159,160], 3D model [102,113,114,115,161] were used in the research for dental diseases diagnostic on the 3D dental model. The studies on the dental disease’s diagnostic on the CBCT dental model used the CT images [124], 3D CT scans [131], CT datasets [162], CBCT and panoramic radiographs [135,136], CBCT images [125,126,127,129,130,133,139,163,164,165], 3D CBCT images [166], CBCT datasets [128,167] and CBCT scans [132,134,137,138,168,169], whereas EDR [120,121,122] images were used to evaluate DL techniques in the studies of dental disease diagnostics through the EDR model. Table 11 shows the images that used to evaluate DL techniques.

### 3.4. What Are the Performance Measurement Techniques Used to Measure DL Techniques?

Every DL pipeline has performance measurements. The model was evaluated on the test dataset of photos after training and validation. Visualization, prediction, and decision making are the key roles in an efficient and effective system. Approximately 56 studies were evaluated to determine the performance measurement techniques used to evaluate DL techniques in the dental practice. Table 12 shows various types of performance measures in different researches studied in this SLR where ‘✓’ indicates that the measure was used by the researcher to evaluate the performance whereas "✗" indicates that the measure was dropped by the researcher. However, accuracy, sensitivity, specificity, precision, recall, and F1 score are still the most used performance measurement in most of the studies. Model performance techniques are shown in Table 12.

## 4. Discussion

For this SLR, relevant papers (papers published related to DL in dentistry) were picked from the databases Scopus, Web of Science, Springer, ACM Digital Library, IEEE Explorer, and Science Direct. To conduct this review, we chose research spanning 2017 to 2022 depending on their popularity. The study was carried out to advocate a systematic review process that would aid future researchers in determining the general framework of a DL-based dental diagnostic. Approximately 48 research papers were studied to answer questions related to DL techniques that are used in different fields of DI using different types of images and performance measurement to evaluate DL techniques. DL, which represents AI, is applied in a variety of societal contexts, including the medical and dental industries, to address real-world problems. The advancement of DL is being ramped up by the advent of self-learning back-propagation techniques which enhance data outputs and process technology in small, gradual changes. As the precision of DL algorithms in healthcare continues to improve, we should expect to see more teamwork in computer-assisted diagnosis. The development of AI-based dental applications is absolutely intriguing. Despite the fact that DL has been found to have potential uses in dentistry in several studies, these systems are still far from being able to replace dental experts. Rather, AI should be considered as a supplementary asset that may help dentists and experts. To guarantee that humans retain the capacity to supervise treatment and make educated decisions in dentistry, it is critical to ensure that DL is incorporated in a safe and regulated manner. The route to effective DL integration in dentistry will need dental and continuing education training, a task that most institutions are now unprepared to meet. MR is a novel phrase that blends features of generative DL, VR, and AR into computer-superimposed information overlays into computer-generated data patches for enhanced teaching and preoperative scheduling. First findings from the multiple DL systems being developed for different areas of dentistry are promising, suggesting that reinforcement learning has a promising future in the dental treatment field. DL technologies have shown potential as a valuable tool for oral health practitioners.

The main focus of the research was on the DL that are used in the dental practice. The study focuses on the various DL techniques (such as ANN, CNN, and GAN) and the applications of DL (such as CAD/CAM, 3D printing, CBCT etc.) utilized in dental procedures. Endodontics, orthodontics, implants, oral surgery, oral medicine, periodontology, zirconium crowns, fixed bridges, dental implant restorations, orthodontic devices, and DI are among the topics covered in the study. (inlays, veneers, onlays), and removable dentures (partial and/or complete) where DL techniques were applied. Moreover, the study determines the type of images required to evaluate the DL practices in dentistry. The performance measurement techniques discussed determine how the researchers can benefit from measures such as accuracy, F1 score, ROC, precision, and recall. The ability of CNNs to recognize and identify anatomical features has showed promise. Some have been taught to recognize and classify teeth from periapical radiographs, for example. Dentists have also employed CNNs to identify and diagnose dental caries. Deep CNNs offer a lot of promise for enhancing the sensitivity of dental caries’ detection, and this, along with their speed, makes them one of the most useful tools in this field. ANNs offer a lot of promise in terms of assisting in clinical decision-making. To get predictable outcomes for patients, it is critical to schedule orthodontic treatments thoroughly. Teeth extractions, on the other hand, are not commonplace as part of an orthodontic treatment plan. As a result, before beginning irreversible operations, it is critical to make the best clinical judgement possible. In individuals with malocclusion, an ANN was used to evaluate if tooth extraction was necessary before orthodontic therapy. Early identification and diagnosis of oral lesions is critical in dental offices because early detection improves prognosis greatly. Because certain oral lesions are precancerous or cancerous, it is critical to obtain an accurate diagnosis and treat the patient appropriately. CNN is beneficial for diagnosing head and neck lesions.

In dentistry, DL has a higher diagnostic accuracy than medical imaging techniques like X-ray or computerized temography (CT) scans for diagnosing oral disorders. Several publications that use DL to treat oral problems have been published in recent years. DL systems can handle the complexities and challenges of autonomous oral disease diagnosis. To date, many review studies on the identification and categorization of oral illnesses have been completed, but only a few of them can provide a clear roadmap for future researchers. Despite the fact that these papers provided a thorough review of the literature on dental diseases and applications, they may have overlooked a few DL concerns.

The preponderance of dental review studies focused on traditional ML or ANNs, which employ feature extraction for diagnosis. They couldn’t handle existing DL architectures for identifying dental diagnosis, such as GCNs, GANs, and ELMs. Although a few review articles on dentistry medical imaging techniques and digital dental technologies have been published, they would be unable to include all imaging modalities used in the identification and categorization of dental diseases. This study lays a strong platform for a comprehensive and critical examination of existing DL-based digital dentistry technologies and dental disease identification.

### 4.1. Contribution

DI is a fairly inexperienced area with a lot of potential for using computer and information sciences to improve dentistry, teaching, management and research. DI encompasses more than just the use of computers in dentistry. This paper provides an in-depth look into deep-neural networks used to detect dental problems. Based on the findings and subsequent discussion, researchers and dentists who work with vast collections of dental pictures, files, and databases will benefit from the knowledge provided by this SLR. As previously indicated, the most recent ML and DL approaches might be useful in identifying, segmenting, categorising, and visualising dental illnesses that are present in a variety of forms. Performance measuring methods will also help in the choice of the best methods, tools, models, and frameworks.

It was expected from the SLR that researchers who would like to work on medical image classification with an emphasis on DL-based dental diagnostics utilizing a range of medical images will find this review informative.

### 4.2. Implications for Practice

In terms of diagnosing oral disorders, prescribing, indications, and contraindications of certain pharmaceuticals in patients with certain circumstances. DI has several clinical uses and instruments. DI provides possible treatments for various dental disorders, but it has limits. Its progress will need individual and profession-wide efforts. Digital dental technology has had a significant impact on how dentists approach patients and how they build novel and complete restorative treatments. Based on the findings and subsequent discussion, researchers and dentists who work with vast collections of dental pictures and files will benefit from the knowledge provided by this SLR. As previously indicated, the most recent ML and DL approaches might be useful in identifying, segmenting, categorizing, and visualizing dental illnesses that are present in a variety of forms. Performance measuring methods will also help in the choice of the best methods, tools, models, and frameworks. In comparison with previous surgical approaches, this methodology offered substantial simplicity and improvements, boosting implant location accuracy while also enhancing patient comfort and compliance. Modern digital technologies have the ability to change dentistry on an educational and clinical level by utilizing AR, VR, and MR to improve students’ learning and clinical training. Both their academic and practical knowledge might grow for the students. These technologies might be useful to dental professionals in their work.

## 5. Limitations and Future Recommendations

Because DL is a keyword used in many applications, data accessibility and domain context are the SLR’s two biggest limitations. Due to the paper’s acceptance and publication period being between 2017 and 2021, certain pertinent articles were not readily available. The selection of English-language studies is a minor restriction. The following are our opinions on the potential applications of DL in DI.

- **The need to collect and annotate a dental image dataset.** When compared to other imaging fields, the dentistry industry finds it difficult to obtain annotated data, which is necessary for DL applications. Dental data annotation is costly, laborious, and time consuming because it requires professionals to devote a lot of their time to it. In addition, it might not always be achievable in unusual circumstances.
- **Advancement in DL methods.** For supervised DL, the majority of DL algorithms rely on annotated data. To address the issue of enormous data unavailability, the supervised DL industry needs switch from being supervised to unsupervised or semi-supervised. How useful can unsupervised and semi-supervised methods be in medicine as a result, and how can we move from supervised learning to transform learning without sacrificing accuracy while keeping in mind how sensitive healthcare systems are?
- **Implementation of AR or VR applications in various fields of dental medicine and education.** By testing the capabilities of AR and VR virtually before putting them to use on patients, dental surgeons and trainees can gain knowledge and confidence in their abiilty to use these methods. Although several research studies have highlighted important limits for users working with haptics, dental surgeions and trainees can perform common and difficult treatments quickly and efficiently by using real-time haptic feedback on virtual patients [170]. There are several potential uses for AR and VR in dentistry, including specialized dental fields, dental education and training, oral and maxillofacial surgery, and pediatric dentistry.
- **Implement an automated tooth disease diagnosis system based on DL methods.** The dental care industry has been severely affected by the COVID-19 pandemic [171]. Indeed, it is necessary to progress an automated method for scanning teeth, identifying, categorizing, and diagnosing dental diseases.
- **Structuring electronic dental records through DL for a clinical decision support system.** Medical informatics’ fundamental and difficult objective is to extract information from unstructured clinical text [172].
- **GNN-based approach.** The most commonly used methods in the DL disciplines is the GNN. By using this method, it is possible to create artificial data that resembles real data nearly exactly and to comprehend the links (i.e., visual relationships) between them, which aids in the identification and classification of dental illnesses. Therefore, GNN might be a great option for handling data ambiguity.

## 6. Conclusions

The current study reviewed all of the noteworthy publications on DL methods for healthcare analysis in this publication. Based on the area of DI, we suggested a classification strategy for arranging the current articles and highlighted numerous significant research prototypes. We also talked about the benefits and drawbacks of applying DL methods to DI. We also discuss some of the most urgent open issues and promising future developments. In recent decades, research on DL and healthcare analysis has been particularly popular. Each year, a large number of new emerging models and developing procedures are created. We believe that this study can give readers a thorough grasp of the important elements of this discipline, explain the most significant developments, and cast some light on potential future research.

## Figures and Tables

**Table 1:** Inclusion and exclusion criteria description of research studies.

ID	Keywords
1	(“Data Learning” OR “DL”) AND (“Dental Informatics” OR “DI”) AND (“Image Data” OR “Dental Data”)
2	(“Data Learning” OR “DL”) AND (“Dental Informatics” OR “DI” OR “Dentistry”) AND (“Image Data” OR “Dental Data”)
3	(“Data Learning” OR “DL”) AND (“Dental Informatics” OR “DI” OR “Dental”) AND (“Image Data” OR “Dental Data”)
4	(“Data Learning” OR “DL”) AND (“Dental Informatics” OR “DI” OR “Dentist”) AND (“Image Data” OR “Dental Data”)

**Table 2:** RQ Selected Studies.

RQ	Studies
Deep learning techniques	40
Dental informatics using deep learning	39
Images to evaluate deep learning techniques	73
Performance measurement techniques to evaluate the deep learning techniques	56

**Table 3:** ANN selected studies.

Authors Name and Year	Methods	Results	Authors Suggestions/Conclusions
Faria et al., (2021) [41]	Custom-made ANN	Detect accuracy = 98.8%, predict accuracy = 99.2%, AUC= 0.9886, 0.9869	This approach may be beneficial for detecting and predicting the RRC’s development in other photos.
Li et al., (2021) [42]	DeepLabv3+, Xception and MobileNetV2	AUC = 0.7, precision = 0.606, recall = 0.415, mIOU = 0.650	Small dataset was used and data augmentation cannot overcome all biases present in small dataset.
Geetha et al., (2020) [43]	Customized BPNN	Accuracy = 97.1%, false positive (FP) rate = 2.8%, ROC area = 0.987, PRC area = 0.987	High quality datasets and improved algorithm can demonstrate good results towards dental practice.
Zanella-Calzada et al., (2018) [44]	Customized ANN	Accuracy = 0.69, AUC values = 0.69 and 0.75	This model can help dentists by providing an easy, free and fast tool for the diagnosis of DC.
Rochman et al., (2018) [46]	ELM	Low error rate = 0.0426	ELM is a powerful predictive tool.
Li et al., (2018) [47]	HMI and ELM	Sensitivities of incisors, canine, premolar, and molars were 78.25 ± 6.02%, 78.00 ± 5.99%, 79.25 ± 7.91%, and 78.75 ± 5.17%	Compared to the ANN approach, this method had a greater classification.
Lu et al., (2018) [48]	PCA and ELM	Accuracy = 79.75%	They are not able to detect the correct name for each landmark, especially for the teeth with similar teeth anatomy.
Li et al., (2018) [49]	GLCM, ELM	Sensitivity= 72%, specificity= 70%, accuracy= 71%	This method is more sensitive and accurate than the wavelet energy and naïve Bayes classifier.

**Table 4:** RNN Selected Studies.

Authors Name and Year	Methods	Results	Authors Suggestions/Conclusions
Alarifi and AlZubi, (2018) [51]	MSGSRNN	Accuracy = 99.25%, sensitivity = 97.63%, specificity = 98.28%	Outlined methodology analyzes patient characteristics and aids to know the failure and success rate of the process of implant treatment
Kumari et al., (2022) [52]	M–ResneXt–RNN, HSLnSSO algorithm	Accuracy = 93.67, sensitivity = 94.66, specificity = 92.73, precision = 92.44, FPR = 7.27, FNR = 5.34, NPV = 94.88, FDR = 7.56, F1-Score = 93.54, MCC = 87.35	Difficult to distinguish tiny items and produces rather coarse characteristics.
Singh and Sehgal, (2021) [53]	customized CNN-LSTM	Accuracy = 96%	This model gets lower performance using large datasets.

**Table 5:** CNN selected studies.

Authors Name and Year	Methods	Results	Authors Suggestions/Conclusions
Prajapati et al., (2017) [16]	Transfer learning with VGG16 pre-trained model	Accuracy = 88.46%	Transfer learning with the VGG16 pre-trained model achieved better accuracy.
Lee et al., (2018) [56]	Pre-trained GoogLeNet Inception v3 network	Accuracy of 89%, 88%, and 82% was observed in the premolar, molar, and both the premolar-molar regions.	In terms of diagnosing dental caries, Deep CNN algorithms are anticipated to be among the best and most productive technique.
Vinayahalingam et al., (2021) [57]	CNN MobileNet V2	Accuracy = 0.87, sensitivity = 0.86, specificity = 0.88, AUC = 0.90	This method forms a promising foundation for the further development of automatic third molar removal assessment.
Choi et al., (2018) [63]	Customized CNN	F1max = 0.74, FPs = 0.88	This system can be used to detect proximal dental caries on several periapical images.
Lee et al., (2021) [65]	Deep CNN (U-Net)	Precision = 63.29%, recall = 65.02%, F1-score = 64.14%	Clinicians should not wholly rely on AI-based dental caries detection results, but should instead use them only for reference.
Yang et al., (2018) [67]	Customized CNN	F1 score = 0.749	The method doesn’t always work on images of molars.
Lee et al., (2018) [68]	Pre-trained deep CNN (VGG-19) and self-trained network	Premolars (accuracy = 82.8%), molars (accuracy = 73.4%)	Using a low-resolution dataset can reduced the accuracy of the diagnosis and prediction of PCT.
Al Kheraif et al., (2019) [69]	Hybrid graph-cut technique and CNN	Accuracy = 97.07%	The DL with convolution neural network system effectively recognizes the dental disease.
Murata et al., (2019) [70]	Customized AlexNet CNN	Accuracy = 87.5%, sensitivity = 86.7%, specificity = 88.3%, AUC = 0.875	The AI model can be a supporting tool for inexperienced dentists.
Krois et al., (2019) [72]	Custom-made CNN	Accuracy = 0.81, sensitivity = 0.81, Specificity = 0.81	ML-based models could minimize the efforts.
Zhao et al., (2020) [77]	Customized Two-staged attention segmentation network	Accuracy = 96.94%, dice = 92.72%, recall = 93.77%	Failure to properly divide the foreground image into teeth areas due to inaccurate pixel segmentation.
Fariza et al., (2020) [78]	U-Net convolution network	Accuracy = 97.61%	Segmentation with the proposed U-Net convolution network results in fast segmentation and smooth image edges.
Lakshmi and Chitra, (2020) [79]	Sobel edge detection with deep CNN	Accuracy = 96.08%	Sobel edge detection with deep CNN is efficient for cavities prediction compared to other methods.
Khan et al., (2021) [80]	U-Net + Densenet121	mIoU = 0.501, Dice coefficient = 0.569	DL can be a viable option for segmentation of caries, ABR, and IRR in dental radiographs.
Moran et al., (2020) [81]	Pre-trained ResNet and an Inception model	Accuracy = 0.817, precision = 0.762, recall = 0.923, specificity = 0.711, negative predictive = 0.902	Clinically, the examined CNN model can aid in the diagnosis of periodontal bone deterioration during periapical examinations.
Chen et al., (2021) [82]	Customized Faster R-CNN	Precision = 0.5, recall = 0.6	Disease lesions with too small sizes may not be indications for faster R-CNN.
Lin and Chang, (2021) [84]	ResNet	Accuracy = 93.33%	In the second stage, endodontic therapy is the most vulnerable to incorrect labeling.
Zhang et al., (2022) [85]	Customized multi-task CNN	Precision = 0.951, recall = 0.955, F-score = 0.953	The method can provide reliable and comprehensive diagnostic support for dentists.
Yu et al., (2020) [91]	Customized ResNet50-FPN	Accuracy = 95.25%, sensitivity = 89.83%, specificity = 96.10%	Only implement caries detection for First Permanent Molar not all teeth.
Rana et al., (2017) [92]	Customized CNN	AUC = 0.746, precision = 0.347, recall = 0.621	Dental professionals and patients can benefit from automated point-of-care early diagnosis of periodontal diseases provided.
Tanriver et al., (2021) [94]	Multiple pre-trained NNs; EfcientNet-b4 architecture	sensitivity = 89.3, precision = 86.2, F1 = 85.7	The suggested model shows significant promise as a low-cost, noninvasive tool to aid in screening procedures and enhance OPMD identification.
Schlickenrieder et al., (2021) [95]	pre-trained ResNeXt-101–32x8d	accuracy = 98.7%, AUC = 0.996	More training is needed in AI-based detection, classification of common and uncommon dental disorders, and all types of restorations.
Takahashi et al., (2021) [96]	YOLO v3 and SSD	mAP = 0.80, mIoU = 0.76	This method was limited accuracy in identifying tooth-colored prosthese.

**Table 6:** GAN selected studies.

Authors Name and Year	Methods	Results	Authors Suggestions/Conclusions
Kim et al., (2020) [99]	CNN, GLCIC, Edge Connect	Improvement of 0.004 mm in the tooth segmentation	The segmentation approach for complete arch intraoral scan data is efficient, time-saving, and as accurate as a manual segmentation method.
Kokomoto et al., (2021) [100]	PGGAN	*p* value < 0.0001	The quantity of trained photos has a significant impact on PGGAN’s ability to generate realistic visuals.

**Table 7:** GNN selected studies.

Authors Name and Year	Methods	Results	Authors Suggestions/ Conclusions
Zhang et al., (2021) [102]	PointNet, DGCNN, PointNet++, PointCNN, MeshSegNet	Accuracy = 95.25, mIoU = 88.99	TSGCNet cannot robustly handle special cases with 12 teeth.
Zheng et al., (2022) [103]	Modified Dynamic Graph CNN (DGCNN)	mIoU = 97.49, accuracy = 98.94	The proposed teeth segmentation is robust to rotten, missing, crowded, and ectopic-tooth cases.

**Table 8:** CAD–CAM selected studies.

Authors Name and Year	Methods	Results	Authors Suggestions/Conclusions
Adel et al., (2018) [111]	SVM, ORB	Accuracy = 92.8%	Regarding the detection of oral epithelial dysplasia, this approach had the highest success rates.
Chatterjee et al., (2018) [112]	SVM, k nearest neighbor, random forest.	Accuracy = 90%	Predictive classifiers are better able to distinguish between illness and control groups when statistical and cytomorphometric features are combined.
Xu et al., (2018) [114]	Customized CNN	Accuracy = 99.06%	It directly satisfies the industrial clinical treatment demands and is also robust to any possible foreign matters on dental model surface.
Tian et al., (2019) [115]	Sparse voxel octree and 3D CNN	Accuracy = 95.96%	the proposed method has great application potential in the computer-assisted orthodontic treatment diagnosis.

**Table 9:** EDR selected studies.

Authors Name and Year	Methods	Results	Authors Suggestions/Conclusions
Cui et al., (2021) [120]	Extreme Gradient Boost (XGBoost) algorithm	Accuracy = 96.2, Precision = 86.5, Recall = 83.0	ML methods showed promise for forecasting multiclass issues, such as varying therapies depending on EDRs.
Kang et al., (2022) [121]	RF, ANN, CNN, GBDT, SVM, LR, LSTM	Accuracy = 92%, F1-score = 90%, precision = 94%, recall = 87%	ML is strongly recommended as a decision-making aid for dental practitioners in the early diagnosis and treatment of tooth caries
Chen, (2021) [122]	NLP	F1-score 83% and 88%	The NLP workflow might be used as the initial stage to training data-based models with structured data.

**Table 10:** CBCT selected studies.

Authors Name and Year	Methods	Results	Authors Suggestions/Conclusions
Miki et al., (2017) [124]	AlexNet network	Accuracy = 91.0%	Automated filling of dental data for forensic identification can benefit from the suggested tooth categorization approach.
Sorkhabi and Khajeh, (2019) [125]	Customized 3D CNN	Hexagonal prism (precision = 84.63%), cylindrical voxel shapes (precision = 95.20%)	This method may help the dentists in the implant treatment from diagnosis to surgery.
Jaskari et al., (2020) [126]	Customized FCDNN	DSC were 0.57 (SD = 0.08) for the left canal and 0.58 (SD = 0.09) for the right canal	Automated DL neural network-based system when applied to CBCT scans can produce high quality segmentations of mandibular canals.
Kwak et al., (2020) [127]	2D SegNet, 2D and 3D U-Nets	2D U-Net (accuracy = 0.82), 2D SegNet (accuracy = 0.96), 3D U-Net (accuracy = 0.99)	With the help of DL, a dentist will be able to create an automated method for detecting canals, which will considerably improve the effectiveness of treatment plans and the comfort of patients.
Kim et al., (2020) [129]	CNN-based DL models	Accuracy = 93%	This method aims at assisting orthodontist to determine the best treatment path for the patient be it orthodontic or surgical treatment or a combination of both.
Orhan et al., (2020) [130]	U-Net	Accuracy = 92.8%	AI systems based on DL methods can be useful in detecting periapical pathosis in CBCT images for clinical application.
Cui et al., (2019) [131]	Customized 3D CNN	DSC = 92.37%, DA = 99.55%, FA = 96.85%	The segmentation will fail when there is extreme gray scale value in CT image and if the tooth has the wrong orientation.
Chen et al., (2020) [132]	Multi-task 3D FCN combined with MWT	Dice = 0.936 (±0.012), Jaccard index = 0.881 (±0.019)	The multi-task 3D FCN combined with MWT can segment individual tooth of various types in dental CBCT images.
Lee et al., (2020) [133]	Fully automated CNN-based U-Net structure	Dice = 0.935, Recall = 0.956, Precision = 0.915	Some portions of the wisdom teeth were usually undetected.
Wang et al., (2021) [134]	Customized CNN	Dice similarity coefficient = 0.934 ± 0.019	DL has the potential to accurately and simultaneously segment jaw and teeth in CBCT scans.
Hiraiwa et al., (2019) [135]	AlexNet and GoogleNet	Accuracy = 86.9%	The deep learning system showed high accuracy in the differential diagnosis of a single or extra root in the distal roots of mandibular first molars.
Lee et al., (2020) [136]	GoogLeNet Inception-v3 architecture	Sensitivity = 96.1%, specificity = 77.1%, AUC = 0.91	Deep CNN architecture trained with CBCT images achieved higher diagnostic performance than that trained with panoramic images.
Ezhov et al., (2021) [137]	Customized CNN	The sensitivity values for aided and unaided groups were 0.8537 and 0.7672 while specificity was 0.9672 and 0.9616 respectively.	The proposed AI system significantly improved the diagnostic capabilities of dentists.
Qiu et al., (2021) [138]	Customized CNN	Dice (%) = 95.29	This model can be viewed as a training goal for a particular application.

**Table 11:** Types of images to evaluate DL techniques.

Image Type	No. of Studies	Studies References
Radiographic images	25	Faria et al., (2021) [41], Geetha et al., (2020) [43], Lee et al., (2018) [68], Prajapati et al., (2017) [16], Choi et al., (2018) [63], Lee et al., (2018) [56], Yang et al., (2018) [67], Al Kheraif, (2019) [69], Murata et al., (2019) [70], Krois et al., (2019) [72], Ekert et al., (2019) [71], Verma et al., (2020) [73], Zhao et al., (2020) [77], Mahdi et al., (2020) [75], Fariza et al., (2020) [78], Lakshmi and Chitra, (2020) [79], Moran et al., (2020) [81], Muresan et al., (2020) [74], Lakshmi and Chitra, (2020) [76], Cantu et al., (2020) [64], Khan et al., (2021) [80], Vinayahalingam et al., (2021) [157], Lee et al., (2021) [65], Chen et al., (2021) [82], Kabir et al., (2021) [83], Lin and Chang, (2021) [84], Zhang et al., (2022) [85], Imak et al., (2022) [87]
NILT	3	Casalegno et al., (2019) [88], Schwendicke et al., (2020) [89], Holtkamp et al., (2021) [90]
Intraoral images	11	Rana et al., (2017) [92], Moutselos et al., (2019) [93], Welikala et al., (2020) [158], Yu et al., (2020) [91], Schlickenrieder et al., (2021) [95], Hossam et al., (2021) [86], Saini et al., (2021) [66], Takahashi et al., (2021) [96], Askar et al., (2021) [97], Goswami et al., (2021) [159], Shang et al., (2021) [160]
3D Model	5	Xu et al., (2018) [114], Tian et al., (2019) [115], Yamaguchi et al., (2019) [113], Cui et al., (2021) [161], Zhang et al., (2021) [102]
CT/CBCT images	26	Miki et al., (2017) [124], Roy et al., (2018) [140], Cui et al., (2019) [131], Phanijjiva et al., (2018) [143], Huang et al., (2021) [162], Hiraiwa et al., (2019) [135], Lee et al., (2020) [136], Sorkhabi and Khajeh, (2019) [125], Jaskari et al., (2020) [126], Kim et al., (2020) [129], Kwak et al., (2020) [127], Orhan et al., (2020) [130], Chung et al., (2020) [163], Lee et al., (2020) [133], Wang et al., (2021) [134], Zheng et al., (2020) [164], Kurt Bayrakdar et al., (2021) [165], Ezhov et al., (2021) [137], Jang et al., (2021) [166], Qiu et al., (2021) [138], Sherwood et al., (2021) [139], Shaheen et al., (2021) [168], Alsomali et al., (2022) [167], Cipriano et al., (2022) [128], Liu et al., (2022) [169], Chen et al., (2020) [132]
EDRs	3	Cui et al., (2021) [120], Kang et al., (2022) [121], Chen et al., (2021) [122]

**Table 12:** Performance measures of deep learning methods.

Study	Accuracy	Precision	Recall	F1 score	Sensitivity	Specificity	FP	AUC	ROC	PRC	mIOU	FPR	NPV	FNR	mAP	IOU	FDR	MCC	dice	DSC	DA	FA	Jaccard
De Araujo Faria et al., (2021) [41]	✓	✗	✗	✗	✗	✗	✗	✓	✗	✗	✗	✗	✗	✗	✗	✗	✗	✗	✗	✗	✗	✗	✗
Li et al., (2021) [42]	✗	✓	✓	✗	✗	✗	✗	✓	✗	✗	✓	✗	✗	✗	✗	✗	✗	✗	✗	✗	✗	✗	✗
Geetha et al., (2020) [43]	✓	✗	✗	✗	✗	✗	✓	✗	✓	✓	✗	✗	✗	✗	✗	✗	✗	✗	✗	✗	✗	✗	✗
Zanella-Calzada et al., (2018) [44]	✓	✗	✗	✗	✗	✗	✗	✓	✗	✗	✗	✗	✗	✗	✗	✗	✗	✗	✗	✗	✗	✗	✗
Li et al., (2018) [47]	✗	✗	✗	✗	✓	✗	✗	✗	✗	✗	✗	✗	✗	✗	✗	✗	✗	✗	✗	✗	✗	✗	✗
Lu et al., (2018) [48]	✓	✗	✗	✗	✗	✗	✗	✗	✗	✗	✗	✗	✗	✗	✗	✗	✗	✗	✗	✗	✗	✗	✗
Li et al., (2018) [49]	✓	✗	✗	✗	✓	✓	✗	✗	✗	✗	✗	✗	✗	✗	✗	✗	✗	✗	✗	✗	✗	✗	✗
Alarifi and AlZubi, (2018) [51]	✓	✗	✗	✗	✓	✓	✗	✗	✗	✗	✗	✗	✗	✗	✗	✗	✗	✗	✗	✗	✗	✗	✗
Kumari et al., (2022) [52]	✓	✓	✗	✓	✓	✓	✗	✗	✗	✗	✗	✓	✓	✓	✗	✗	✓	✓	✗	✗	✗	✗	✗
Singh and Sehgal, (2021) [53]	✓	✗	✗	✗	✗	✗	✗	✗	✗	✗	✗	✗	✗	✗	✗	✗	✗	✗	✗	✗	✗	✗	✗
Prajapati et al., (2017) [16]	✓	✗	✗	✗	✗	✗	✗	✗	✗	✗	✗	✗	✗	✗	✗	✗	✗	✗	✗	✗	✗	✗	✗
Lee et al., (2018) [56]	✓	✗	✗	✗	✗	✗	✗	✗	✗	✗	✗	✗	✗	✗	✗	✗	✗	✗	✗	✗	✗	✗	✗
Vinayahalingam et al., (2021) [57]	✓	✗	✗	✗	✓	✓	✗	✓	✗	✗	✗	✗	✗	✗	✗	✗	✗	✗	✗	✗	✗	✗	✗
Choi et al., (2018) [63]	✗	✗	✗	✓	✗	✗	✓	✗	✗	✗	✗	✗	✗	✗	✗	✗	✗	✗	✗	✗	✗	✗	✗
Lee et al., (2021) [65]	✗	✓	✓	✓	✗	✗	✗	✗	✗	✗	✗	✗	✗	✗	✗	✗	✗	✗	✗	✗	✗	✗	✗
Yang et al., (2018) [67]	✗	✗	✗	✓	✗	✗	✗	✗	✗	✗	✗	✗	✗	✗	✗	✗	✗	✗	✗	✗	✗	✗	✗
Lee et al., (2018) [68]	✓	✗	✗	✗	✗	✗	✗	✗	✗	✗	✗	✗	✗	✗	✗	✗	✗	✗	✗	✗	✗	✗	✗
Al Kheraif et al., (2019) [69]	✓	✗	✗	✗	✗	✗	✗	✗	✗	✗	✗	✗	✗	✗	✗	✗	✗	✗	✗	✗	✗	✗	✗
Murata et al., (2019) [70]	✓	✗	✗	✗	✓	✓	✗	✓	✗	✗	✗	✗	✗	✗	✗	✗	✗	✗	✗	✗	✗	✗	✗
Krois et al., (2019) [72]	✓	✗	✗	✗	✓	✓	✗	✗	✗	✗	✗	✗	✗	✗	✗	✗	✗	✗	✗	✗	✗	✗	✗
Zhao et al., (2020) [77]	✓	✗	✓	✗	✗	✗	✗	✗	✗	✗	✗	✗	✗	✗	✗	✗	✗	✗	✓	✗	✗	✗	✗
Fariza et al., (2020) [78]	✓	✗	✗	✗	✗	✗	✗	✗	✗	✗	✗	✗	✗	✗	✗	✗	✗	✗	✗	✗	✗	✗	✗
Lakshmi and Chitra, (2020) [79]	✓	✗	✗	✗	✗	✗	✗	✗	✗	✗	✗	✗	✗	✗	✗	✗	✗	✗	✗	✗	✗	✗	✗
Khan et al., (2021) [80]	✗	✗	✗	✗	✗	✗	✗	✗	✗	✗	✓	✗	✗	✗	✗	✗	✗	✗	✓	✗	✗	✗	✗
Moran et al., (2020) [81]	✓	✓	✓	✗	✗	✓	✗	✗	✗	✗	✗	✗	✓	✗	✗	✗	✗	✗	✗	✗	✗	✗	✗
Chen et al., (2021) [82]	✗	✓	✓	✗	✗	✗	✗	✗	✗	✗	✗	✗	✗	✗	✗	✗	✗	✗	✗	✗	✗	✗	✗
Lin and Chang, (2021) [84]	✓	✗	✗	✗	✗	✗	✗	✗	✗	✗	✗	✗	✗	✗	✗	✗	✗	✗	✗	✗	✗	✗	✗
Zhang et al., (2022) [85]	✗	✓	✓	✓	✗	✗	✗	✗	✗	✗	✗	✗	✗	✗	✗	✗	✗	✗	✗	✗	✗	✗	✗
Yu et al., (2020) [91]	✓	✗	✗	✗	✓	✓	✗	✗	✗	✗	✗	✗	✗	✗	✗	✗	✗	✗	✗	✗	✗	✗	✗
Rana et al., (2017) [92]	✗	✓	✓	✗	✗	✗	✗	✓	✗	✗	✗	✗	✗	✗	✗	✗	✗	✗	✗	✗	✗	✗	✗
Tanriver et al., (2021) [94]	✗	✓	✗	✓	✓	✗	✗	✗	✗	✗	✗	✗	✗	✗	✗	✗	✗	✗	✗	✗	✗	✗	✗
Schlickenrieder et al., (2021) [95]	✓	✗	✗	✗	✗	✗	✗	✓	✗	✗	✗	✗	✗	✗	✗	✗	✗	✗	✗	✗	✗	✗	✗
Takahashi et al., (2021) [96]	✗	✗	✗	✗	✗	✗	✗	✗	✗	✗	✓	✗	✗	✗	✓	✗	✗	✗	✗	✗	✗	✗	✗
Zhang et al., (2021) [102]	✓	✗	✗	✗	✗	✗	✗	✗	✗	✗	✓	✗	✗	✗	✗	✗	✗	✗	✗	✗	✗	✗	✗
Zheng et al., (2022) [103]	✓	✗	✗	✗	✗	✗	✗	✗	✗	✗	✓	✗	✗	✗	✗	✗	✗	✗	✗	✗	✗	✗	✗
Adel et al., (2018) [111]	✓	✗	✗	✗	✗	✗	✗	✗	✗	✗	✗	✗	✗	✗	✗	✗	✗	✗	✗	✗	✗	✗	✗
Chatterjee et al., (2018) [112]	✓	✗	✗	✗	✗	✗	✗	✗	✗	✗	✗	✗	✗	✗	✗	✗	✗	✗	✗	✗	✗	✗	✗
Xu et al., (2018) [114]	✓	✗	✗	✗	✗	✗	✗	✗	✗	✗	✗	✗	✗	✗	✗	✗	✗	✗	✗	✗	✗	✗	✗
Tian et al., (2019) [115]	✓	✗	✗	✗	✗	✗	✗	✗	✗	✗	✗	✗	✗	✗	✗	✗	✗	✗	✗	✗	✗	✗	✗
Cui et al., (2021) [120]	✓	✓	✓	✗	✗	✗	✗	✗	✗	✗	✗	✗	✗	✗	✗	✗	✗	✗	✗	✗	✗	✗	✗
Kang et al., (2022) [121]	✓	✓	✓	✓	✗	✗	✗	✗	✗	✗	✗	✗	✗	✗	✗	✗	✗	✗	✗	✗	✗	✗	✗
Chen, (2021) [122]	✗	✗	✗	✓	✗	✗	✗	✗	✗	✗	✗	✗	✗	✗	✗	✗	✗	✗	✗	✗	✗	✗	✗
Miki et al., (2017) [124]	✓	✗	✗	✗	✗	✗	✗	✗	✗	✗	✗	✗	✗	✗	✗	✗	✗	✗	✗	✗	✗	✗	✗
Sorkhabi and Khajeh, (2019) [125]	✗	✓	✗	✗	✗	✗	✗	✗	✗	✗	✗	✗	✗	✗	✗	✗	✗	✗	✗	✗	✗	✗	✗
Jaskari et al., (2020) [126]	✗	✗	✗	✗	✗	✗	✗	✗	✗	✗	✗	✗	✗	✗	✗	✗	✗	✗	✗	✓	✗	✗	✗
Kwak et al., (2020) [127]	✓	✗	✗	✗	✗	✗	✗	✗	✗	✗	✗	✗	✗	✗	✗	✗	✗	✗	✗	✗	✗	✗	✗
Kim et al., (2020) [129]	✓	✗	✗	✗	✗	✗	✗	✗	✗	✗	✗	✗	✗	✗	✗	✗	✗	✗	✗	✗	✗	✗	✗
Orhan et al., (2020) [130]	✓	✗	✗	✗	✗	✗	✗	✗	✗	✗	✗	✗	✗	✗	✗	✗	✗	✗	✗	✗	✗	✗	✗
Cui et al., (2019) [131]	✗	✗	✗	✗	✗	✗	✗	✗	✗	✗	✗	✗	✗	✗	✗	✗	✗	✗	✗	✓	✓	✓	✗
Chen et al., (2020) [132]	✗	✗	✗	✗	✗	✗	✗	✗	✗	✗	✗	✗	✗	✗	✗	✗	✗	✗	✓	✗	✗	✗	✓
Lee et al., (2020) [133]	✗	✓	✓	✗	✗	✗	✗	✗	✗	✗	✗	✗	✗	✗	✗	✗	✗	✗	✓	✗	✗	✗	✗
Wang et al., (2021) [134]	✗	✗	✗	✗	✗	✗	✗	✗	✗	✗	✗	✗	✗	✗	✗	✗	✗	✗	✓	✗	✗	✗	✗
Hiraiwa et al., (2019) [135]	✓	✗	✗	✗	✗	✗	✗	✗	✗	✗	✗	✗	✗	✗	✗	✗	✗	✗	✗	✗	✗	✗	✗
Lee et al., (2020) [136]	✗	✗	✗	✗	✓	✓	✗	✓	✗	✗	✗	✗	✗	✗	✗	✗	✗	✗	✗	✗	✗	✗	✗
Ezhov et al., (2021) [137]	✗	✗	✗	✗	✓	✗	✗	✗	✗	✗	✗	✗	✗	✗	✗	✗	✗	✗	✗	✗	✗	✗	✗
Qiu et al., (2021) [138]	✗	✗	✗	✗	✗	✗	✗	✗	✗	✗	✗	✗	✗	✗	✗	✗	✗	✗	✓	✗	✗	✗	✗

## Data Availability

Not applicable.

## Supplementary Materials

The following supporting information can be downloaded at: https://www.mdpi.com/article/10.3390/healthcare10101892/s1.

## Abbreviations

The following abbreviations are used in this manuscript:

Abbreviation	Full Name
SLR	Systematic Literature Review
DL	Deep Learning
DI	Dental Informatics
MI	Medical Informatics
IT	information technology
CAD	Computer-aided design
CAM	computer-aided manufacturing
CBCT	Cone Beam Computed Tomography
CT	Computerized Tomography
EDR	Electronic Dental Record
EHR	Electronic Health Records
FEA	Finite Element Analysis
FEM	Finite Element Method
VR	Virtual Reality
AR	Augmented Reality
ML	Machine Learning
ANN	Artificial Neural Network
CNN	Convolutional Neural Network
GAN	Generative Adversarial Network
ELM	Extreme Learning Machines
GCN	Graph Convolutional Network
RNN	Recurrent Neural Network
RRC	Radiation-Related Caries
NILT	Near-infrared Light Transillumination
PCT	Periodontally Damaged Teeth
pBL	Periodontal Bone Loss
SVM	Support Vector Machine
DPR	Dental Panoramic Radiograph
TI	Transillumination
R-CNN	Region-based Convolutional Neural Network
GNN	Graph Neural Network
FP	False Positive
ROC	Receiver Operating Characteristic
PRC	precision recall curve
AUC	Area Under Curve
mIOU	mean Intersection over Union
NPV	Negative Predictive Value
mAP	Mean Average Precision
IOU	Intersection Over Union
DSC	Dice similarity coefficient
DA	Detection Accuracy
FA	identification accuracy
FPR	false-positive predictions
MI-DCNNE	multi-input deep convolutional neural network ensemble
ICDAS	International Caries Detection and Assessment System
DNN	Deep Neural Network
UCDA	novel caries detection and assessment
TSGCNet	two-stream graph convolutional network
PGGAN	progressive growing of generative adversarial networks
SLFNs	Single-Hidden Layer Feed forward Neural Networks
LSTM	Long Short-Term Memory
SSD	Single Shot MultiBox Detector
BPNN	Back-Propagation Neural Network
