# Supplementary material for: Analysis of Deep Learning Techniques for Dental Informatics: A Systematic Literature Review
Source: Healthcare (Basel). 2022 Sep 28;10(10):1892. doi: 10.3390/healthcare10101892 (PMC9602147; doi:10.3390/healthcare10101892)
Supplement: Supplementary file 1 [file healthcare-10-01892-s001.zip › healthcare-1868711-supplementary.pdf]

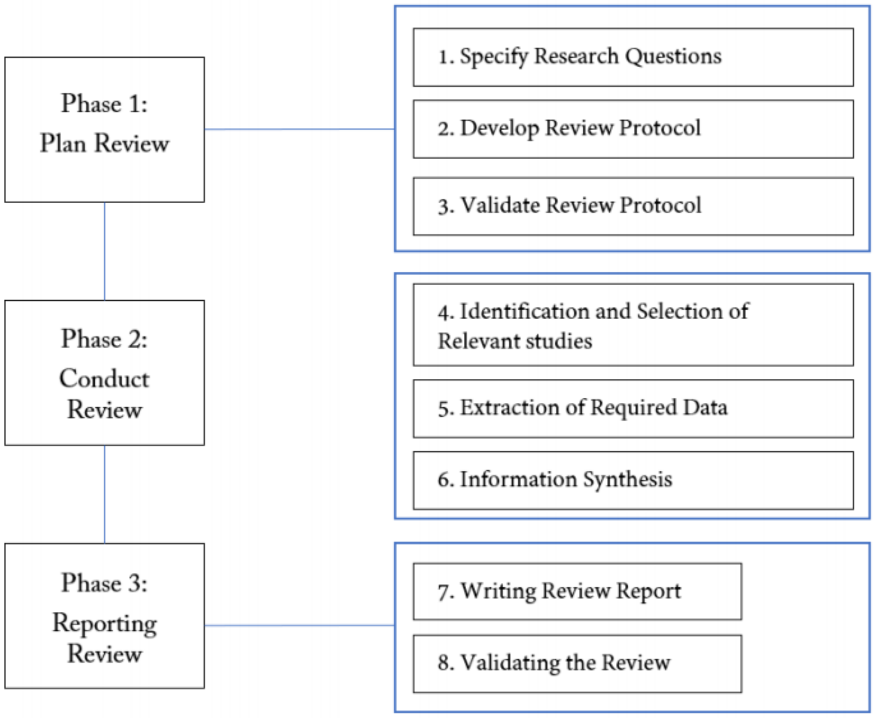

Figure S1. Phase Involved in SLR process

Table S1. (a) Inclusion criteria description of research studies

Inclusion Criteria
The research was relevant to data informatics sources.
The research was directly related to the images and data used in dental practices.
The research was conducted using DL techniques for DI applications.
The research used performance measurement techniques.
The research was conducted for the analysis of deep learning techniques performance in dental objects.
For duplicate publications of the same study, the newest and most complete one was selected.
This is recorded for only one study whose related work appeared two times.

Table S1. (b) exclusion criteria description of research studies.

Exclusion Criteria
Studies that were irrelevant to dental illness and the dental healthcare domain were skipped.
Studies with the word “informatics” were used in many domains which are excluded due to limitations of our scope.

Table S2. Quality checklist.

No.	Questions
1	Was there a strong focus on dental informatics, such as CAD/CAM, in the studies?
2	Was the study able to describe how deep learning is applied in the field of dental informatics?
3	Is there a model to evaluate deep learning approaches in dental informatics that has been proposed?
4	Is the study concentrating on the basic deep learning approaches for huge dental practice data?
5	Is there any mention of model performance adopting core approaches in the study?

Table S3. Data extraction.

Study
Study Research Problem Contributions
RQ1: Deep learning
RQ2: Dental informatics
RQ3: Images and Datasets
RQ4: Performance Measurement Techniques

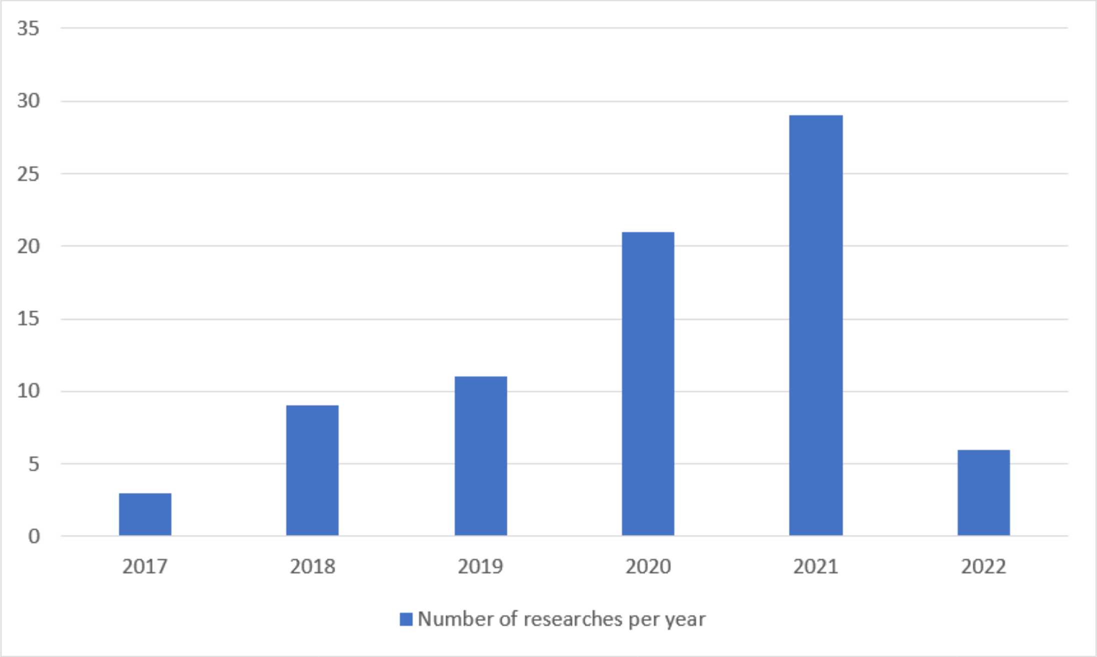

Figure S2. This figure shows the published number of deep-learning-based dental informatics studies in the past 5 years and the current year.
